# Risk factors for insufficient weight and height gain in children with congenital heart disease followed up at a nutrition outpatient clinic

**DOI:** 10.1590/1984-0462/2022/40/2020512IN

**Published:** 2022-05-27

**Authors:** Beatriz Cassaniga Talassi, Tulio Konstantyner, Stela de Almeida Miranda, Heitor Pons Leite

**Affiliations:** a Universidade Federal de São Paulo, São Paulo, SP, Brazil.

**Keywords:** Heart defects, congenital, Pediatrics, Growth, Risk factors, Anthropometry, Follow-up studies, Cardiopatias congênitas, Pediatria, Crescimento, Fatores de risco, Antropometria, Seguimentos

## Abstract

**Objective:**

To describe weight and height evolution and to identify risk factors for insufficient anthropometric growth in children with congenital heart disease.

**Methods:**

Historical cohort study including 131 children with congenital heart disease, followed up at a nutrition outpatient clinic. The anthropometric indices over time (initial score, after 12 and 24 months of follow-up) were analyzed using generalized estimating equations. The outcome was ‘insufficient weight-height gain’, defined as an increase of ≤0.5 in the z-score of weight-for-age (W/A), height-for-age (H/A) or body mass index-for-age (BMI/A) after 12 months of follow-up. Multiple logistic regression models were applied to identify risk and confounding factors.

**Results:**

The z-scores of W/A (p<0.001) and BMI/A (p<0.001) improved after 12 months, as well as the three indexes after 24 months (p<0.001). At the end of this period, 55.7% of the patients did not achieve an increase of >0.5 in the Z score of W/A; 77.1%, of H/A; and 45.8%, of BMI/A. A follow-up of less than five appointments was associated with insufficient gain in W/A (OR 7.78; 95%CI 3.04–19.88), H/A (OR 10.79; 95%CI 2.22–52.45) and BMI/A (OR 2.54; 95%CI 1.12–5.75). Not having undergone cardiac surgery and being aged ≥12 months were factors associated with insufficient W/A gain (OR 3.95; 95%CI 1.38–11.29/OR 3.60; 95%CI 1.33–9.72) and BMI/A (OR 2.81; 95%CI 1.08–7.28/OR 3.39; 95%CI 1.34–8.56). Low income was associated with insufficient H/A gain (OR 4.11; 95%CI 1.25–13.46).

**Conclusions:**

Being aged less than or 12 months, the lowest number of appointments, absence of surgical treatment and low family income were risk factors for insufficient weight and height gain in children with congenital heart disease.

## Resumo

**Objetivo:**

Descrever a evolução pôndero-estatural e identificar fatores de risco para o crescimento antropométrico insuficiente de crianças com cardiopatia congênita.

**Métodos:**

Coorte histórica de 131 crianças com cardiopatia congênita acompanhadas em ambulatório de nutrição clínica. Os escores dos índices antropométricos ao longo do tempo (inicial, após 12 e 24 meses de acompanhamento) foram analisados por equações de estimativas generalizadas. O desfecho foi o ganho pôndero-estatural insuficiente, definido como aumento ≤0,5 nos escores Z de peso para idade (P/I), estatura para idade (E/I) e índice de massa corpórea para idade (IMC/I) em 12 meses de acompanhamento. Foram aplicados modelos de regressão logística múltipla para identificação dos fatores de risco e controle dos fatores de confusão.

**Resultados:**

Os escores Z de P/I (p<0,001) e IMC/I (p<0,001) melhoraram após 12 meses, e os três índices após 24 meses (p<0,001). Ao final desse período, 55,7% dos pacientes não alcançaram aumento >0,5 escore Z de P/I; 77,1%, de E/I; e 45,8%, de IMC/I. O acompanhamento inferior a cinco consultas associou-se ao ganho insuficiente de P/I (OR 7,78, IC95% 3,04–19,88), E/I (OR 10,79, IC95% 2,22–52,45) e IMC/I (OR 2,54, IC95% 1,12–5,75). Não ter sido submetido à cirurgia cardíaca e ter idade ≥12 meses estiveram associados ao ganho insuficiente de P/I (OR 3,95, IC95% 1,38–11,29/ OR 3,60, IC95% 1,33–9,72) e IMC/I (OR 2,81, IC95% 1,08–7,28/OR 3,39, IC95% 1,34–8,56), e a baixa renda, ao ganho insuficiente de E/I (OR 4,11, IC95% 1,25–13,46).

**Conclusões:**

Idade ≥12 meses, menor número de consultas, ausência de tratamento cirúrgico e baixa renda familiar foram fatores de risco para a evolução pôndero-estatural insuficiente em crianças com cardiopatia congênita.

## INTRODUCTION

Congenital heart diseases are characterized by structural or functional abnormalities of the heart and/or major intrathoracic vessels. The global prevalence of congenital heart diseases is of eight to ten children per one thousand live births^1^ and represents the main cause of morbidity and mortality caused by congenital malformations, especially in the early neonatal period.^2^ In Brazil, the underreporting of cases of congenital heart disease and the inexistence of reliable statistics make it difficult to estimate its prevalence.^3^

Children with heart disease often present with changes in growth due to multifactorial causes. There is higher metabolic demand and more oxygen consumption because of increased myocardial effort, respiratory muscles and the hematopoietic system, and there is a reduction in the intake of energy due to fatigue, anorexia and early satiety.^4^ These factors, in different levels, reduce the availability of energy to meet the necessary demand for physical growth.^5^ After birth, linear growth and total body mass reduce at the ratio of the heart disease severity.^6^ Therefore, identifying the risk factors for insufficient weight and height gain may prevent or avoid the deterioration of malnutrition.

Studies that performed a longitudinal assessment of children with congenital heart disease described growth throughout the first years of life, and investigated factors associated with growth deficit and low weight gain in these patients.^6–15^ Most studies evaluated the effect of surgery on growth.^7,8,9,10,11,15^ Despite the relevant role of surgery in the anthropometric recovery of children with congenital heart disease, in relation to other factors, a variable percentage of them does not present improvements in anthropometric indicators, which is usually referred to as growth catch-up. One of these is the epidemiological factor, which has been little studied as a predictor of nutritional recovery in patients with congenital heart disease.

This study aimed at describing weight and height evolution, and at identifying risk factors for its insufficient evolution in children with congenital heart disease and low socioeconomic status, followed-up at an interdisciplinary nutrition outpatient clinic.

## METHOD

This is a historical cohort study of a 24-month follow-up including children with congenital heart disease in an interdisciplinary nutrition outpatient clinic. The study was approved by the Research Ethics Committee at Universidade Federal de São Paulo (Unifesp), Report n. 2.748.948 and Protocol n. 0456/2018.

All patients were assessed and monitored in four-week or shorter intervals, depending on clinical status, by an interdisciplinary team composed of cardiologists, pediatricians, nutritionists, psychologists, speech and language therapists, and social workers according to the nutrition outpatient clinic support provided to the children with heart disease.^16^ Body measurements and adherence to diet were verified after each visit, and macro and micronutrients were supplemented when necessary. The patients who could not meet the requirements of oral intake received enteral nutrition tube feeding.

In this study, 168 children were eligible and aged less than 10 years; they did not present with edema and were followed-up between January, 2002, and April, 2019. Thirty-seven attended only the first appointment or presented with incomplete anthropometric data, which resulted in a sample loss of 22% for the analysis of anthropometric evolution, which was performed with 131 children.

The anthropometric indices of the patients were assessed in three moments throughout the studied period (M0: initial; M1: after 12 months; and M2: after 24 months of follow-up). The considered outcome was insufficient weight and height gain, defined by an increase ≤0.5 of the Z-score values of anthropometric indices in up to 12 months of follow-up: weight for age (W/A), height for age (H/A), and body mass index for age (BMI/A). In this study, the Z-score >0.5 cut off point was adopted for considering the minimum expected gain for the 12-month outpatient clinic follow-up.

The following variables were considered as explanatory: sex, age at first appointment, type of heart disease (cyanogenic or acyanogenic), ROSS classification,^17^ palliative or corrective cardiac surgery, pulmonary hypertension, genetic syndrome, gestational age and weight at birth, number of siblings, family income (in number of minimum wages), maternal schooling, number of appointments and nutritional status at birth and at admission.

The data were collected from medical records. Body weight was measured using a Filizola® electronic pediatric scale, with 0.005 kg accuracy for children aged less than two years. For the others, we used a Filizola® platform digital scale, with 0.1 kg accuracy. Length for children aged less than two years was measured using a horizontal anthropometer. For those aged more than two years, height was measured using a Filizola® stadiometer fixated to the wall, with a 0.1cm accuracy. The anthropometric measurements were verified once and taken by two nutritionists, who differed throughout time. In spite of that, the measurements were only taken after training, according to the standardized protocol of execution of the care unit.

Nutritional status was classified according to the guidelines by the World Health Organization (WHO) (2006/2007).^18^ Patients whose Z-score of at least one of the indicators (W/A, H/A and BMI/A) was lower than -2 were considered as malnourished. For premature infants, we considered the corrected age until the age of 2. For children with Down syndrome, we used specific Brazilian growth curves. Z-scores were calculated using the WHO software, Anthro, version 1.0, and WHO Anthro Plus, version 3.2.2.

The continuous variables were expressed as median and interquartile interval, and categorical variables were shown as absolute and percentage distribution. Generalized estimating equation (GEE) with gaussian distribution were used to analyze the anthropometric indices in the three moments (initial, after 12 and 24 months of follow-up). The chi-squared test was used to compare categorical variables. To identify the risk factors for insufficient weight and height gain, as well as the control of confounding variables, three multiple logistic regression models were adjusted. The criterion used to include the explanatory variables in the models was p value ≤0.20 in the univariate analysis. The significance level was set to 0.05. We investigated terms of interaction and possible collinearity between the variables that remained significant in the final models. The quality of the adjustment of the logistic regression models was assessed by the Hosmer-Lemeshow test. Receiver Operating Characteristics Curve (ROC curves) of the explanatory models for the outcome were developed considering the three analyzed anthropometric indices. Statistical analysis was performed using the Stata software for Statistics and Data Science, version 14.0.

## RESULTS

Table 1 shows the demographic, socioeconomic, neonatal and clinical/anthropometric characteristics of the 168 children studied at admission.

**Table 1.** Demographic, socioeconomic, neonatal and clinical/anthropometric characteristics of children with congenital heart disease admitted at an interdisciplinary nutrition outpatient clinic.

Characteristics	n	Category	Value
Demographic			
Male gender [n (%)]	168	Yes	84 (50.0)
Age (months) [M (IQR)]			7 (3.7–14.6)
Age (<24 months) [n (%)]	168	Yes	141 (83.9)
Socioeconomic [n (%)]			
Family income (<3 minimum wages)	157	Yes	122 (77.7)
Maternal schooling: incomplete elementary school	160	Yes	29 (18.1)
Only child	165	Yes	59 (35.8)
Neonatal			
Type of birth [n (%)]	165	C-section	83 (50.3)
		Natural	77 (46.7)
		Forceps	5 (3.0)
Weight at birth (<2500g) [n (%)]	165	Yes	41 (24.8)
Gestational age at birth (<37 weeks) [n (%)]	166	Yes	35 (21.1)
Anthropometric indices at birth (Z score) [M (IQR)]	163	WAZ	-0.75 (-1.88; 0.01)
	148	HAZ	-1.52 (-2.23; -0.47)
	148	BMIAZ	-0.02 (-0.91; 0.68)
Clinic/anthropometric			
Cyanogenic heart disease [n (%)]	167	Yes	84 (50.3)
ROSS classification [n (%)]	164	1	139 (84.8)
		2	10 (6.1)
		3	14 (8.5)
		4	1 (0.6)
Pulmonary hypertension [n (%)]	164	Yes	18 (11.0)
Genetic syndrome [n (%)]	168	Yes	44 (26.2)
Delay in NPMD [n (%)]	164	Under investigation	5 (3.0)
		Yes	67 (40.9)
		No	97 (59.1)
Cardiac Surgery [n (%)]	168	Yes	122 (72.6)
Anthropometric indices at admission (Z score) [M (IQR)]	168	WAZ	-2.67 (-3.92; -1.72)
	168	HAZ	-2.33 (-3.22; -1.39)
	168	BMIAZ	-2.07 (-3.15; -1.10)

M: median; IQR: interquartile range; NPMD: neuropsychomotor development; WAZ: Z-score of weight-for-age; Z-L/A: Z-score of length-for-age; BMIAZ: Z-score of body mass index-for-age; HAZ: Z-score of height-for-age.

The most common malformations were: interventricular (41.7%) and interatrial (28.6%) communication, arterial duct persistence (20.2%), pulmonary stenosis (12.5%) and hypoplastic left heart syndrome (9.5%). Of the total, 72.6% (n=122) individuals presented with multiple cardiac malformations.

Cardiac surgery was corrective in 90 patients, and performed before the first appointment in 93. The median number of appointments after 12 and 24 months of follow-up was 5 and 7, respectively. The appointments took place, in average, every 2.4 months in the first and every 3.4 months in the second year of follow-up. During the study, 8.3% of the patients died (n=14).

Of the 168 eligible patients, 80.4% had at least one anthropometric index <-2, and 46.7% showed alterations in all indices. Low weight was observed in 67.9% of the individuals, and low height in 61.3%; thinness was observed in 53.6% of the children.

**Figure 1.**
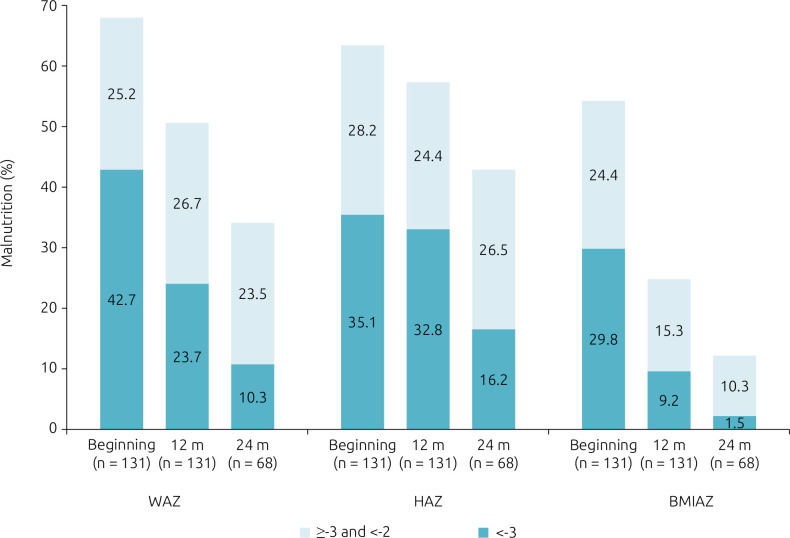
Prevalence of malnutrition (Z-score ≥-3 and <-2) and severe malnutrition (Z-score <-3), according to anthropometric indices (weight-for-age, height-for-age, and body mass index-for-age) at the beginning and after 12 and 24 months of follow-up.

**Figure 2.**
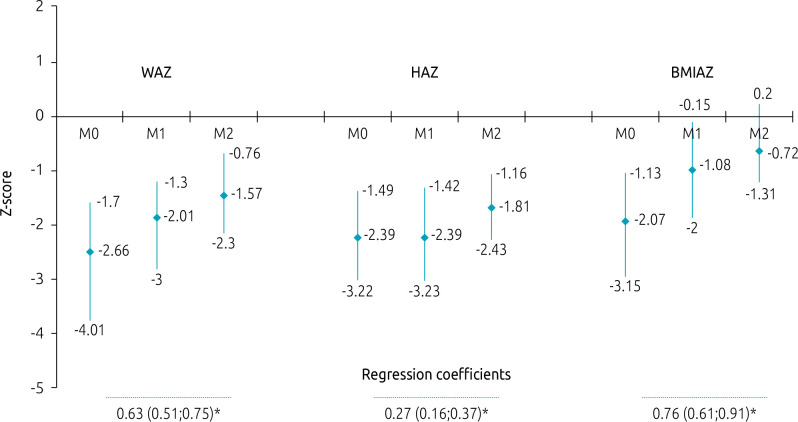
Medians and interquartile intervals of the Z-scores of anthropometric indices (weight-for-age, height-for-age, and body mass index-for-age) and regression coefficients in relation to moments of follow-up.

WAZ: Z-score of weight-for-age; HAZ: Z-score of height-for-age; BMIAZ: Z-score of body mass index-for-age; M0 (n=131): moment of admission; M1 (n=131): moment after 12 months of follow-up; M2 (n=68): moment after 24 months of follow-up. Regression coefficients based on generalized estimating equation (GEE) models (0 to 24 months); *p<0.001.

Of the 131 children followed-up as to anthropometric evolution, 81.7% were malnourished considering at least one of the three anthropometric indices in the beginning of follow-up — 51 were malnourished considering all of the indices. Generally, after 12 months, the proportion of severe malnutrition decreased in 20.6, 19.0 and 2.3%, according to BMIAZ, WAZ, and HAZ, respectively. Of the 68 patients who were still followed-up after 24 months, 42.7% presented with some level of height deficit, and 33.8%, with W/A deficit. Figure 1 shows the distribution of each anthropometric index in the three moments of the study, according to the level of malnourishment. The anthropometric variations observed in the three follow-up moments are presented in Figure 2.

W/A: weight-for-age; H/A: height-for-age; BMI/A: body mass index-for-age.

There was significant improvement in anthropometric scores WAZ and BMIAZ in the 12-month period, which was not true for HAZ, whose regression coefficient was 0.05 (-0.11–0.2; p=0.562). In the 24-month period, this improvement occurred in the three anthropometric indices.

Of the 131 followed-up for 12 months, 55.7% (n=73) did not reach an increase higher than Z-score 0.5 for WAZ; 77.1% (n=101) for HAZ; and 45.8% (n=60) for BMIAZ. In 24 months, of the 68 children who were still followed-up, 30.9% (n=21) did not reach an increase higher than Z-score 0.5 for WAZ; 39.7% (n=27) for HAZ; and 35.3% (n=24) for BMIAZ.

The risk factors for gain ≤0,5 of WAZ, HAZ, and BMIAZ during the 12 months of follow-up that remained in the logistic models are described in Table 2. For each outcome, a multiple logistic regression analysis model was adjusted and included sex, type of heart defect and specific Z-score of the anthropometric index at admission.

**Table 2.** Adjusted prevalence rates and odds ratio and the respective confidence intervals (95%) of the risk factors for insufficient weight and height gain (≤0.5 Z-score), in 12 months of outpatient follow-up.

Associated factors		Gain ≤0,5 In Z-score		OR (95%CI)	p-value
		%	n		
WAZ (n=131)					
Age (months)	≥12	72.2	(26/36)	3.60 (1.33–9.72)	0.011
	<12	49.5	(47/95)	1.00	
Heart surgery	No	77.1	(27/35)	3.95 (1.38–11.29)	0.010
	Yes	47.9	(46/96)	1.00	
N. of appointments	<5	82.3	(42/51)	7.78 (3.04–19.88)	<0.001
	≥5	38.7	(31/80)	1.00	
HAZ (n=122)					
Family income (n. of minimum wages)	<3	82.1	(78/95)	4.11 (1.25–13.46)	0.020
	≥3	63.0	(17/27)	1.00	
N. of appointments	<5	94.1	(48/51)	10.79 (2.22–52.45)	0.003
	≥5	66.2	(53/80)	1.00	
BMIAZ (n=131)					
Age (months)	≥12	66.7	(24/36)	3.39 (1.34–8.56)	0.010
	<12	37.9	(36/95)	1.00	
Heart surgery	No	60.0	(21/35)	2.81 (1.08–7.28)	0.034
	Yes	40.6	(39/96)	1.00	
N. of appointments	<5	60.8	(31/51)	2.54 (1.12–5.75)	0.025
	≥5	36.2	(29/80)	1.00	

OR: *Odds Ratio*; 95%CI: 95% confidence interval; WAZ: Z-score of weight-for-age; HAZ: Z-score of height-for-age; BMIAZ: Z-score of body mass index for age. Logistic models adjusted for sex, type of heart defect and Z-score of anthropometric indices at admission.

There was no collinearity or interaction between the variables that remained in the adjusted models. Type of surgery, ROSS classification, pulmonary hypertension, genetic syndrome, gestational age at birth, weight at birth, maternal schooling, being an only child and anthropometric score at birth were not eligible to compose the final model.

**Figure 3.**
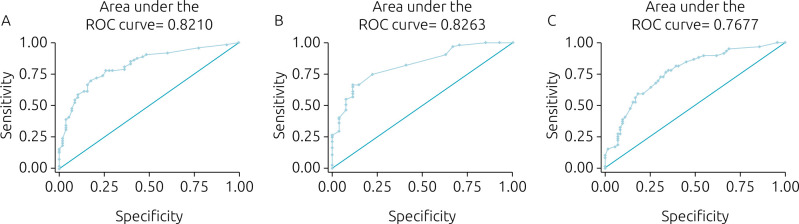
ROC (*Receiver Operating Characteristics Curves*) curves of the explanatory models for the insufficient weight and height evolution in the period of 12 months of follow-up for (A) Z-score of weight-for-age, (B) Z-score of height-for-age, and (C) Z-score of body mass index-for-age.

Figure 3 shows the ROC curves of the explanatory models for the outcome in the 12-month follow-up period considering WAZ, HAZ, and BMIAZ. The areas under the curve of the models for a Z-score gain ≤0.5 were the following: WAZ 0.82; HAZ: 0.83; and BMIAZ: 0.77.

ROC: *Receiver Operating Characteristics Curve*.

Of the analyzed children, regarding insufficient anthropometric growth, 68 were submitted to Cardiac surgery before the first appointment, being the median time of surgery of 1.8 month (minimum of 0.6 and maximum of 68 months before the first appointment). Twenty-seven underwent surgery after the first appointment, with median time of 4.2 months (minimum of 0.5 and maximum of 22 months after the initial appointment). Of the surgeries performed before the first appointment, 54 (79.4%) were corrective, and among those that occurred during follow-up, 22 (81.5%).

Table 3 presents the comparison of prevalence rates of insufficient weight and height gain (Z-score ≤0.5) of three groups classified according to the moment (before or during the 12-month follow-up), or the non-performance of the surgery.

**Table 3.** Prevalence rates and their respective confidence intervals (95%) of insufficient weight and height gain (Z-score ≤0.5), according to the moment (before or during the 12-month follow-up) or the non-performance of surgery (n=131).

Anthropometric index (gain ≤0.5)	All (n=131)	Performance of surgery			p-value*
		Before follow-up (n=68)	During follow-up (n=27)	No surgery (n=36)	
WAZ	55.7 (47.00–64.10)	50.0 (38.10–61.90)	40.7 (23.80–60.20)	77.8 (61.00–88.70)	0.005
HAZ	71.1 (69.00–83.60)	72.1 (60.10–81.50)	74.1 (54.10–87.40)	88.9 (73.40–95.90)	0.139
BMIAZ	45.8 (37.40–54.50)	38.2 (27.40–50.40)	44.4 (26.80–63.60)	61.1 (44.20–75.30)	0.083

WAZ: Z-score of weight-for-age; HAZ: Z-score of height-for-age; BMIAZ: Z-score of body mass index-for-age; *p-value based on the chi-squared test. Values expressed in prevalence and 95% confidence interval.

## DISCUSSION

In this study, four out of five children with congenital heart disease presented with moderate to severe malnutrition in the beginning of follow-up. Approximately half of them presented Z-scores of the three anthropometric indices below -2, and two thirds had low height. The high prevalence of malnutrition is in accordance with that observed in two other studies carried out in countries in the same stage of development (85 and 90.4%)^10,19^, in contrast with that of developed countries (15%).^20^

In 12 months of follow-up, approximately half of the children presented with insufficient evolution of BMIAZ (45,8%) and WAZ (55.7%), and three quarters of HAZ (77.1%). There was significant increase of BMIAZ and WAZ after 12 and 24 months, which occurred for HAZ only after 24 months.

The lowest number of appointments in the first year of follow-up was associated with the insufficient anthropometric evolution of the three anthropometric indices. Not undergoing surgery and being aged more than 12 months or 12 months were associated with insufficient gain of WAZ and BMIAZ; family income lower than three minimum wages was associated with insufficient gain of HAZ.

Body weight deficit, which shows acute malnourishment, occurs earlier than that of height, whose deficit reflects a chronic malnutrition status. The low weight and low height prevalence rates range in relation to different characteristics of the children in the beginning of each study, such as the performance or not of surgery, age group, time of exposure and disease severity.^12,19^

The gain of BMIAZ and WAZ was higher than that of HAZ at the end of the first year of follow-up, because weight gain usually precedes height gain during nutrition recovery. Even though the follow-up time was a factor of improvement for the three indices, the recovery of height requires a longer period of time. Besides, genetic and hormonal factors also play an important role on growth.^21,22^

The lower number of appointments was a risk factor for insufficient gain in the three anthropometric indices. The more frequent outpatient clinic follow-up contributes with an improved nutritional status, for allowing more contact with the care team and, consequently, more chances of creating bonds with the family. It also allows the earlier identification of non-adherence to the conducts, besides the adoption of nutritional guidance that is compatible with the family dynamics.^16,23^

Being aged more than 12 months was associated with insufficient weight gain, expressed by WAZ and A-BMI/A. Likewise, in another study, it was shown that younger children presented with more potential for nutritional recovery and growth (catch-up growth), when compared to older ones.^13^ Besides the fact that hemodynamic disorder is present for longer, the older the age, the slower the anthropometric recovery. Children in the first trimester of life have high growth speed and use 35% of the total energy demand for growth. This proportion is reduced by half at six months of age, and corresponds to only 3% at the end of the first year of life.^24^ However, when indicated, clinical and surgical intervention should take place as soon as possible to prevent the worsening of malnutrition.^25^

The non-performance of surgery was also associated with insufficient weight gain (WAZ and BMIAZ), which can be explained by the permanence of the hemodynamic disorder and its negative impact on nutritional status. Even though post-surgical growth has shown to be related with previous nutritional status, the association we found was independent from the initial anthropometric indices.

The wide variation in the period of time in which children of this study were submitted to surgery may have interfered in the 12-month weight and height evolution, considering that anthropometric recovery is clearer right after surgery. Vaidyanathan et al.^12^ observed that maximum weight and height nutrition recovery takes place in the first postoperative year, becoming stable after this period. This suggests that, with surgical correction, the malnourishment attributed to the direct effects of heart disease is overcome, and, after that, other factors, such as genetic^21^ and dietary ones,^14^ would be associated with recovery.

The delay in growth is not always reversed after corrective surgery. In this study, 29 children (42.7%) remained with low height after two years of follow-up, and 62.1% had already undergone corrective surgery. Of the 43 children who had undergone corrective surgery, 18 (41.9%) remained with height <-2. Likewise, Vaidyanathan et al. noticed that 28.9% of the children remained with low height after the same follow-up time.^12^ Heart disease may reduce bone age due to chronic hypoxemia.^26^ Under conditions of hypoxia, in experimental models, there was stimulation of the formation of osteoclasts and activation of bone reabsorption, which, together with extracellular acidification, led to a reduction in bone density and mineral content.^27^ A study showed children with cyanogenic heart disease who, even after the Fontan procedure, presented with lower corrected bone mass by age and height in adolescence.^28^

Another factor that potentially has an impact on growth is the non-use of nutritional substrates in the phase of the first spurt due to the state of malnutrition or prolonged disease. Children whose growth was damaged for a long period of time during the first years of life do not recover this height deficit, even if the cause is reversed; therefore, they do not reach their genetic potential.^29^

In this study sample, lower income was a risk factor for the insufficient evolution of height. Low family income expresses inadequate diet, household status and basic sanitation, which increases nutritional risk. In a study carried out in Turkey, it was possible to observe nutritional recovery in most infants 12 months after surgical correction, except in a small subgroup of patients with low income.^10^ Vaidyanathan et al. did not observe any association between low socioeconomic status and the permanence of malnutrition in Indian children after 24 months of follow-up.^12^ The discrepancy between results can be explained by the use of different assessment parameters of socioeconomic status, and because low income would not be the only factor to influence post-surgical growth. Once growth deficit is a more common problem in developing countries, socioeconomic status should also be considered in studies that assess factors associated with the evolution of linear growth in children with heart disease.^30^

This study used data from care-related records, which led to the lack of complete information, especially some neonatal anthropometric data. The classification of the initial nutritional status was performed in the first outpatient clinic visit, and not at the time when congenital heart disease was diagnosed. There was no follow-up for 24 months of all patients that were initially eligible, which prevented the assessment of linear growth in a prolonged period. The variability of the time interval between the diagnosis of heart disease and the first appointment, the age of the children and the type of surgery (palliative or corrective) may have interfered in the estimation and the identification of risk factors for the outcome. Besides, the chosen study design made it impossible to analyze the effect of post-surgical time on nutritional recovery.

On the other hand, we included premature infants and those with low weight at birth, which increases the power of generalization of results. The analysis of physical growth evolution during the follow-up was based on Z-score gain of anthropometric indices. In other studies, which included epidemiological factors as potential exposure variables, the anthropometric evolution was defined in a dichotomic manner, based on the final Z score <-2, which restricts the information about evolution only to the classification of the final nutritional status. Besides, multiple logistic models showed good precision to predict insufficient weight and height evolution.

It is possible to conclude that most children had insufficient weight and height evolution after 12 months of follow-up. The lower attendance in appointments, the non-performance of surgery, being aged 12 months or older and low family income were factors that had a negative effect on nutritional recovery, regardless of sex, type of heart defect and initial nutritional status. The existence of an infrastructure of care associated with the use of a planned care protocol, which can facilitate the access to appointments with an interdisciplinary team, allowing the performance of surgery in the proper period of time, could reduce the negative nutritional impact of heart disease, especially among children from low income families. Given the multiple factors potentially associated with the weight and height evolution of children with congenital heart disease, it is recommended that these be investigated together in prospective studies.
